# Association of multimorbidity and changes in health-related quality of life following myocardial infarction: a UK multicentre longitudinal patient-reported outcomes study

**DOI:** 10.1186/s12916-021-02098-y

**Published:** 2021-09-28

**Authors:** T. Munyombwe, T. B. Dondo, S. Aktaa, C. Wilkinson, M. Hall, B. Hurdus, G. Oliver, R. M. West, A. S. Hall, C. P. Gale

**Affiliations:** 1 Leeds Institute of Cardiovascular and Metabolic Medicine, University of Leeds, Leeds, LS2 9JT UK; 2 Leeds Institute for Data Analytics, University of Leeds, Leeds, UK; 3 Department of Cardiology, Leeds Teaching Hospitals NHS Trust, Leeds, UK; 4 Population Health Sciences Institute, Faculty of Medical Sciences, Newcastle University, Newcastle upon Tyne, UK; 5 Lancashire, UK; 6 Leeds Institute of Health Sciences, University of Leeds, Leeds, UK

**Keywords:** Multimorbidity, Health-related quality of life, EQ5D, Myocardial infarction

## Abstract

**Background:**

Multimorbidity is prevalent for people with myocardial infarction (MI), yet previous studies investigated single-health conditions in isolation. We identified patterns of multimorbidity in MI survivors and their associations with changes in HRQoL.

**Methods:**

In this national longitudinal cohort study, we analysed data from 9566 admissions with MI from 77 National Health Service hospitals in England between 2011 and 2015. HRQoL was measured using EuroQol 5 dimension (EQ5D) instrument and visual analogue scale (EQVAS) at hospitalisation, 6, and 12 months following MI. Latent class analysis (LCA) of pre-existing long-term health conditions at baseline was used to identify clusters of multimorbidity and associations with changes in HRQoL quantified using mixed effects regression analysis.

**Results:**

Of 9566 admissions with MI (mean age of 64.1 years [SD 11.9], 7154 [75%] men), over half (5119 [53.5%] had multimorbidities. LCA identified 3 multimorbidity clusters which were severe multimorbidity (591; 6.5%) with low HRQoL at baseline (EQVAS 59.39 and EQ5D 0.62) which did not improve significantly at 6 months (EQVAS 59.92, EQ5D 0.60); moderate multimorbidity (4301; 47.6%) with medium HRQoL at baseline (EQVAS 63.08, EQ5D 0.71) and who improved at 6 months (EQVAS 71.38, EQ5D 0.76); and mild multimorbidity (4147, 45.9%) at baseline (EQVAS 64.57, EQ5D 0.75) and improved at 6 months (EQVAS 76.39, EQ5D 0.82). Patients in the severe and moderate groups were more likely to be older, women, and presented with NSTEMI. Compared with the mild group, increased multimorbidity was associated with lower EQ-VAS scores (adjusted coefficient: −5.12 [95% CI −7.04 to −3.19] and −0.98 [−1.93 to −0.04] for severe and moderate multimorbidity, respectively.

The severe class was more likely than the mild class to report problems in mobility, OR 9.62 (95% confidence interval: 6.44 to 14.36), self-care 7.87 (4.78 to 12.97), activities 2.41 (1.79 to 3.26), pain 2.04 (1.50 to 2.77), and anxiety/depression 1.97 (1.42 to 2.74).

**Conclusions:**

Among MI survivors, multimorbidity clustered into three distinct patterns and was inversely associated with HRQoL. The identified multimorbidity patterns and HRQoL domains that are mostly affected may help to identify patients at risk of poor HRQoL for which clinical interventions could be beneficial to improve the HRQoL of MI survivors.

**Trial registration:**

ClinicalTrials.gov NCT01808027 and NCT01819103

**Supplementary Information:**

The online version contains supplementary material available at 10.1186/s12916-021-02098-y.

## Background

Multimorbidity, the presence of two or more long-term health conditions in an individual, is a major clinical and public health challenge [1, 2]. Around two thirds of patients with cardiovascular diseases have at least one long-term health condition [3]. Co-morbidities are present in 60% of patients hospitalised with myocardial infarction (MI) and are associated with significant years of life lost [4]. Following MI, patients frequently report poor health related quality of life (HRQoL), which may not recover [5], and is independently associated with higher rates of death at 1 year [6, 7]. Poor HRQoL is more commonly reported among women [8, 9], older people with frailty [5, 10], non-white ethnicity [11], and in those that experience bleeding complications relating to dual anti-platelet therapy [12]. Several previous studies have looked at individual chronic health conditions and their impact on health-related quality of life in MI survivors. Patients with long-term health conditions such as hypertension [13], diabetes [14], angina [15], depression [16], and chronic obstructive pulmonary disease (COPD) report poor HRQoL. Specific disease combinations may have greater effects than others on functional, physical and mental status [17], quality of life [18], and mortality [4, 19].

However, there is a paucity of longitudinal studies that have investigated the association of multimorbidity and temporal changes in HRQoL [20–22]. Existing studies are limited by a focus on mortality as the primary end point [4, 21], use of cross-sectional designs [20, 23], and focusing on individual disease in isolation. Different disease combinations may have greater effects than others on patient outcomes. A previous study [4] found an association of combinations of disease clusters with patient survival in MI patients. We have extended this research by looking at how combinations of disease clusters in survivors of MI impact on changes in HRQoL. Notably, there are few large-scale datasets available that combine clinical data with robust evaluation of temporal changes in HRQoL for patients with MI. In this study, we identified baseline multimorbidity patterns, compare changes in HRQoL across multimorbidity clusters, and investigate the associations of these clusters with changes in HRQoL after MI.

## Methods

### Data and participants

#### Setting

We analysed data from Evaluation of the Methods and Management of Acute Coronary Events, EMMACE-3 and EMMACE-4, which are multicentre longitudinal national cohort studies of outcomes following MI combining survey data with national clinical registration data (ClinicalTrials.gov NCT01808027 and NCT01819103) [24]. The study included patients aged 18 years and over who were hospitalized with MI, defined by the third universal definition as either ST-elevation myocardial infarction (STEMI) or non-STEMI (NSTEMI) [25]. Participants were recruited from 77 National Health Service (NHS) hospitals in England between November 1, 2011, and June 24, 2015. They were consented to participate by a trained researcher during their hospital admission (data flow is shown in Additional file 1: Figure S1).

Patients at a terminal stage of any illness, and those for whom follow-up would be inappropriate or impractical, were excluded. Consenting patients were asked to complete a self-administered questionnaire at the time of enrolment in hospital, and at 1, 6, and 12 months following discharge from hospital. This included information about HRQoL assessed using the three level EuroQol 5-dimension (EQ-5D-3L) instrument [24]. For non-responders who were alive and who had not withdrawn from the study, repeat questionnaires were sent by post on up to three occasions before the date of the next follow-up contact. Data for consenting patients were linked to the national clinical register of MI admissions in the UK (Myocardial Ischaemia National Audit Project, MINAP [26]) to gather information about patients medical history including presence of hypertension, diabetes mellitus, angina, asthma or chronic obstructive pulmonary disease (COPD), cerebrovascular disease (CVSD), peripheral vascular disease (PVD), chronic heart failure, chronic renal failure, type of MI (NSTEMI or STEMI), and in-hospital as well as post-discharge treatments and medications.

### Health-related quality of life

The outcome of this study was HRQoL assessed using self-reported EQ-5D-3L [27]. This contains two subscales: a descriptive system (EQ-5D) and a visual analogue scale (EQ-VAS). EQ-5D comprises five dimensions: mobility, self-care, usual activities, pain/discomfort, and anxiety/depression. Each domain has three levels (3L): no problems, some problems, and extreme problems. The EQ-5D-3L dimensions data may be summarised as a single index score ranging from −0.5 to 1, with scores less than 0 indicating states ‘worse than death’, 0 indicating no quality of life, or ‘death’, and 1 indicating full health and therefore no problems in any domain. The index score was standardised to the UK population [28]. The EQ-VAS score ranges from 0 to 100, with 0 denoting the worst and 100 the best, health state imaginable. The EQ-5D questionnaire has previously been validated in patients following MI [29]. A difference in score of 7 for VAS and 0.05 for EQ-5D score are regarded as clinically important [30], and these thresholds were used to define a clinically important change between subgroups.

### Exposure

The exposure was multimorbidity clusters based on 7 pre-existing long-term health conditions recorded in the MINAP registry including hypertension, diabetes mellitus, asthma or chronic obstructive pulmonary disease (COPD), cerebrovascular disease (CVSD), peripheral vascular disease (PVD), chronic heart failure, and chronic renal failure. In the data source used for the study, the Myocardial Ischaemia National Audit Project (MINAP) [26] registry information on multi-morbidities is given as binary variables. No further detail is given beyond this; therefore, we were restricted to use the information as recorded.

### Other variables

Sociodemographic, health characteristics, and clinical variables included age, sex, ethnicity (white vs other), smoking status (never vs ex or current), body mass index (BMI) (kg/m^2^), past medical history of MI, angina, diagnosis (STEMI or NSTEMI), revascularisation (percutaneous coronary intervention [PCI] vs. no PCI; coronary artery bypass graft [CABG] surgery vs no CABG surgery), medications (aspirin, β blockers, statins, and ACE inhibitors), and referral for cardiac rehabilitation (yes/no).

### Statistical analysis

Patient characteristics according to multimorbidity clusters were described using frequencies and percentages for categorical data and for continuous data as means and standard deviation. Chi-square test and ANOVA were used to assess univariate associations between categorical and continuous patients’ characteristics and multimorbidity clusters, respectively. We corrected for multiple testing in the tables using the Hochberg correction, using a false discovery rate of 0.05.

Latent class analysis (LCA) [31] using Mplus software version 8 was used to identify clusters of multimorbidity for 7 pre-existing long-term health conditions recorded in the MINAP registry at hospital admission including hypertension, diabetes mellitus, asthma or chronic obstructive pulmonary disease (COPD), cerebrovascular disease (CVSD), peripheral vascular disease (PVD), chronic heart failure, and chronic renal failure.

Latent class analysis (LCA) is a statistical technique used to determine subgroups within populations that share certain outward characteristics [31]. In LCA, class membership is based on probability of belonging to a class membership given the pattern of responses they have on indicator variables, and there are no clear cut assignments. LCA is a “person-centred” approach of deriving typologies, unlike “variable-centred” tradition that uses arbitrary cut-offs for classifying individual cases [32]. We used LCA instead of cluster analysis [33] because unlike cluster analysis or k-means clustering, LCA is model-based and an evaluation of how well a proposed LCA model represents the data can be conducted [33] using Bayesian information criterion (BIC) [34], Akaike’s information criteria (AIC) [35], and Bootstrap likelihood ratio test (BLRT) [36] *p* values. We fitted several latent class models varying the number of classes up to 5 classes to identify the best class solution with 1000 random starting values each with 100 iterations. Bootstrap *p* values based on 500 replications were used to assess model fit. The model goodness of fit statistics, entropy, classification matrix, class frequencies, and class conditional probabilities is reported in Additional file 1: Table S1, Table S2, and Table S3. The utility of using LCA has been demonstrated by other researchers who used it to identify clinical phenotypes with differential treatment responses [37] and patient outcomes [4, 38, 39].

The optimal LCA model was selected based on Bayesian information criterion (BIC), Akaike’s information criteria (AIC), Bootstrap likelihood ratio test (BLRT) *p* values, and clinical interpretation. For the BIC and AIC the optimal model is the model with the smallest value, and for the BLRT, the optimal number of clusters is where the *p* value becomes non-significant at significance level 0.05. A three-class multimorbidity solution provided the best latent class model fit based on BIC, AIC, and BLRT *p* values. Patient allocation was based on posterior probabilities of belonging to a class. In order to determine the adjusted association of baseline patient characteristics with multimorbidity cluster membership, we fitted multinomial logistic regression models and reported using odds ratios and their corresponding 95% confidence intervals.

Multilevel linear regression analysis [40] of longitudinal changes in EQ-VAS scores (in hospital, 1 month, 6 months, and 12 months) was performed to investigate the associations of multimorbidity clusters and temporal changes in patient perceptions of health. The outcomes data are repeated measurements overtime, and patients are clustered within hospitals; therefore, data are not independent, and a multilevel linear model was used to account for the clustering in the data. The multilevel models were fitted in steps, first an unconditional means model was used to determine the significance of the 2 random-effect terms (hospital and patient). To check whether the linear model was appropriate, we examined the distribution of residuals to check that there were approximately normally distributed. The normal probability plots are reported in Additional file 1: Figure S2. Where the normality assumption was violated, the multilevel Tobit regression [41] models were fitted and compared to the multilevel linear model results and the results were similar. Tobit regression models are commonly used to analyse patient reported outcome measures data with ceiling and floor effects.

The analysis adjusted for time (categorised as baseline, 1 month, 6 months, and 12 months), age, sex, ethnicity (white vs other), smoking status (never vs ex or current), past medical history of MI, angina, diagnosis (STEMI or NSTEMI), revascularisation (percutaneous coronary intervention [PCI] vs. no PCI; coronary artery bypass graft [CABG] surgery vs no CABG surgery), medications (aspirin, β blockers, statins, and ACE inhibitors), referral for cardiac rehabilitation (yes/no), and interactions of time and multimorbidity class. The confounders were selected based on clinical consideration and previous research [4, 42].

Multilevel linear regression analysis of longitudinal changes in EQ-5D scores (in hospital, 1 month, 6 months, and 12 months) was performed to investigate the associations of multimorbidity clusters and temporal changes in HRQoL. Effect sizes (regression coefficients) and their corresponding 95% confidence intervals were used to assess the adjusted magnitude of the difference in EQ-VAS, EQ-5D scores across multimorbidity classes.

To investigate the association of multimorbidity clusters and changes in HRQoL measured by EQ-5D dimensions, five multilevel logistic regression models were fitted for the EQ-5D dimensions (mobility, self-care, activities, pain, and anxiety, and depression) adjusting for age, sex, ethnicity (white versus other) smoking status (never vs ex or current), past medical history of MI, angina, diagnosis (STEMI or NSTEMI), revascularisation (percutaneous coronary intervention [PCI] vs. no PCI; coronary artery bypass graft [CABG] surgery vs no CABG surgery), medications (beta-blockers, statins, angiotensin converting enzymes (ACE), aspirin), cardiac rehabilitation (yes/no), and interactions of time and multimorbidity.

The ‘extreme problem’ category for some domains of the EQ5D measure was endorsed by few individuals for some domains (e.g., self-care and mobility); therefore, we combined the EQ-5D levels ‘some problems’ and ‘extreme problems’ and the responses were binary (no problems vs some/extreme problems) and adjusted odds ratios (OR) and their corresponding 95% confidence intervals were used to assess the adjusted associations of multimorbidity classes and EQ-5D dimensions.

### Missing data

In longitudinal studies, missing data are commonly encountered, subjects can be missed at a particular assessment time; therefore, subjects may provide outcome data at some, but not all study time points resulting in incomplete data. Participants might drop out of the study or could be lost to follow up. In this study, there was missing outcome data over time. A sensitivity analysis was conducted comparing baseline characteristics of patients with complete data and those that dropped out (Additional file 1: Table S4). The drop outs were not significantly different from the followed up subjects in sex, ethnicity, previous angina, chronic renal failure, and PVD but were significantly different in age, diagnosis (NSTEMI, STEMI), Index of Multiple Deprivation (IMD), smoking status, history of previous acute MI (AMI), PCI, prevalence of diabetes, baseline EQ-5D, and EQ-VAS scores. In order to include patients with incomplete outcome data and to mitigate against biases which may arise as a result of such an omission, we used a multilevel model that includes all participants even if they were not assessed at all 4 time points.

All statistical tests were two-sided, and statistical significance was considered at *p* <0.05. Analyses were conducted using stata (IC) version 15

### Patient involvement

Whilst no patients were involved in setting the research question or the study design, we have co-produced this research manuscript with a patient with prior MI who provided input into the interpretation of the research findings, gave a critical review of the manuscript, and will work with our research team in ensuring its widespread dissemination.

## Results

### Patient characteristics

Descriptive statistics for the cohort are shown in Table 1. From 16,780 acute coronary syndrome hospitalisations across the 77 recruiting hospitals in England between 2010 and 2015, we excluded 4250 who did not have a diagnosis of MI and 2964 non-index hospitalisations, leaving an analytical cohort of 9566 patients (3908 STEMI, 5658 NSTEMI, Additional file 1: Figure S1 in the supplement). The EQ-5D-3L questionnaire response rates were 97.5% (9332/9566), 74.7% (6679/8945), 63.9% (5572/8719), and 62.7% (5047/8043) at hospitalisation, 1 month, 6 months, and 12 months, respectively. Reasons for non-participation at each stage included death and withdrawals from the study. Sixty-nine out of 9566 patients died in hospital. There were significant differences in some baseline characteristics between the patients who dropped out and those who completed the study (Additional file 1: Table S4).

**Table 1:** Patient characteristics by multimorbidity class

Variable	Total cohort ***n***=9566	Multimorbidity class 1 (severe) 591 (6.5%)	Multimorbidity class 2 (moderate) 4301 (47.6%)	Multimorbidity class 3 (mild) 4147 (45.9%)	***P*** value^†^
Age, mean (SD)	64.1 (11.9)	74.8 (9.3)	68.8 (10.3)	57.7 (10.5)	<0.001*
Women, *n* (%)	2384 (24.9)	177 (30.0)	1274 (29.7)	803 (19.4)	<0.001*
IMD, mean (SD)	23.0 (15.7)	24.0 (16.2)	22.3 (15.2)	23.7 (16.1)	0.04
BMI, mean(SD)	28.7 (6.0)	29.8 (5.4)	28.9 (5.5)	28.2 (6.5)	<0.001*
Ethnicity (Caucasian), *n* (%)	8136 (85.1)	507 (94.8)	3639 (94.2)	3530 (93.3)	0.41
Ex/current smoking status, *n* (%)	6248 (65.3)	368 (64.6)	2709 (64.2)	5979 (67.2)	<0.001*
NSTEMI, *n* (%)	5658 (59.2)	508 (86.0)	2870 (66.7)	2046 (49.3)	<0.001*
STEMI, *n* (%)	3908 (40.8)	83 (14.0)	1431 (33.3)	2101 (50.7)	
**Co-morbidities**					
Diabetes, *n* (%)	1714 (17.9)	375 (64.7)	1143 (27.1)	107 (2.6)	<0.001*
Peripheral vascular disease, *n* (%)	317 (3.3)	121 (20.5)	168 (3.9)	24 (0.6)	<0.001*
Cerebrovascular disease, *n* (%)	428 (4.5)	122 (20.6)	297 (6.9)	0	<0.001*
Asthma or COPD, *n* (%)	1166 (12.2)	147 (24.9)	651 (15.1)	358 (8.6)	<0.001*
Chronic renal failure, *n* (%)	289 (3.0)	235 (39.8)	45 (1.1)	8 (0.2)	<0.001*
Heart failure, *n* (%)	212 (2.2)	203 (34.4)	0	6 (0.1)	<0.001*
Hypertension, *n* (%)	4078 (42.6)	408 (69.0)	3295 (76.6)	337 (8.1)	<0.001*
Previous MI, *n* (%)	1522 (15.9)	384 (65.0)	1006 (23.4)	114 (2.8)	<0.001*
Angina, *n* (%)	1792 (18.7)	374 (63.3)	1322 (30.7)	80 (1.9)	<0.001*
Previous PCI, *n* (%)	899 (9.4)	169 (28.7)	617 (14.4)	98 (2.4)	<0.001*
CABG surgery, *n* (%)	643 (6.7)	152 (25.8)	452 (10.5)	29 (0.7)	<0.001*
**Discharge medications**					
Aspirin, *n* (%)	8147 (85.2)	464 (79.2)	3626 (84.7)	3636 (87.9)	<0.001*
ACE inhibitors, *n* (%)	7609 (79.5)	418 (71.3)	3389 (79.2)	3436 (83.1)	<0.001*
Beta-blockers, *n* (%)	7592 (79.7)	430 (73.4)	3340 (78. 0)	3439 (83.1)	<0.001*
Statin, *n* (%)	8140 (85.1)	477 (81.4)	3619 (84.6)	3636 (87.9)	<0.001*
**Rehabilitation**					
Cardiac rehabilitation, *n* (%)	8509 (88.9)	502 (85.1)	3854 (90.3)	3877 (94.5)	<0.001*

*IMD* Index of Multiple Deprivation, *BMI* body mass index, *ACE* angiotensin-converting enzyme, *STEMI* ST-elevation myocardial infarction, *NSTEMI* non-STEMI, *CABG* coronary artery bypass grafting, *PCI* percutaneous coronary intervention, *AMI* acute myocardial infarction, *COPD* Chronic Obstructive Pulmonary Disease. ^†^Obtained from chi-squared or analysis of variance (ANOVA) as appropriate. *Significant after Hochberg correction using a false discovery rate of 0.05

Baseline demographic data were missing in less than 5% of cases, except for the Index of Multiple Deprivation (IMD) (55.0%) and ethnicity (9.3%). Overall, 25.1% (2,397) of the analytical cohort were women. The mean age was 64.1 (standard deviation [SD] 11.9) years; mean body mass index (BMI) 28.7 (6.04) kg/m^2^, median IMD 18.5 (interquartile range [IQR] 10.9 to 31.8), and 6248 (65.3%) were smokers or ex-smokers.

### Long-term health conditions in MI patients

Frequently observed long-term health conditions included hypertension 4078 (42.6%), angina 1792 (18.7%), PVD 428 (4.5%), diabetes mellitus 1714 (17.9%), COPD 1166 (12.2%), CVSD 428 (4.5%), chronic renal failure 289 (3.0%), and heart failure 212 (2.2%). Overall, 53.5% of participants (5119) had one or more long-term health conditions; 3562 out of 9566 (37.2%) had none; 3157 (33.0%) had one, 1414 (14.8%) had two, 419 (4.4%) had three, 110 (1.2%) had 4, and 19 (0.2%) had five or more co-morbidities. Co-morbidity data were not recorded in 885 (9.3%) cases. Co-morbidities were more prevalent in women (1442, 60.1%) than men (3674, 51.3%).

### Multimorbidity clusters in MI patients

LCA identified three distinct multimorbidity classes and were clinically labelled as class 1: severe multimorbidity, 591 (6.5%), class 2: moderate multimorbidity, 4301 (47.6%), and class 3: mild multimorbidity, 4147 (45.9%).

**Fig. 1:**
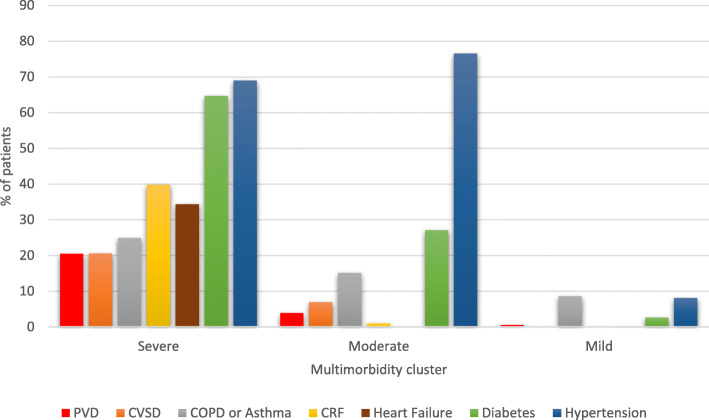
Percent of patients with conditions in each multimorbidity class. Severe multimorbidity patients tended to have high levels of all co-morbidities, moderate multimorbidity patients tended to have hypertension, and diabetes, and mild multimorbidity were patients with few co-morbidities. Note COPD indicates chronic obstructive pulmonary disease; CVSD, cerebrovascular disease; PVD, peripheral vascular disease; CRF, chronic renal failure

The majority of health conditions were more prevalent in the severe class: diabetes (severe 64.7%; moderate 27.1%; and mild 2.6%), PVD (20.5%, 3.9% and 0.6%), CVSD (20.6%, 6.9% and 0%), CRF (39.8%, 1.1% and 0.2%) and heart failure (34.4%, 0.0% and 0.1%), and asthma or COPD (24.9%, 15.1% and 8.6%); however, hypertension was more prevalent in the moderate class (severe 69% moderate 76.6% and mild 8.1%) (Fig. 1).

### Factors associated with multimorbidity class membership

Compared with the mild multimorbidity class, patients in the severe and moderate multimorbidity classes were older (mean age of 74.8, 68.8 vs 57.5 years), had a higher proportion of women (30.0%, 29.7% vs 19.4%), more commonly presented with NSTEMI (86.0%, 66.7% vs 49.3%) and more often had a history of angina (63.3%, 30.7% vs 1.9%), previous MI ( 65.0%, 23.4% vs 2.8%), CABG (25.8%, 10.5% vs 0.7%), and previous PCI (28.7%, 14.4% vs 2.4%). Compared to the severe class, the mild and moderate classes were more likely to have STEMI (50.7% and 33.3% vs. 14.0%) (Table 1).

### Multimorbidity and perceptions of health state

**Fig. 2:**
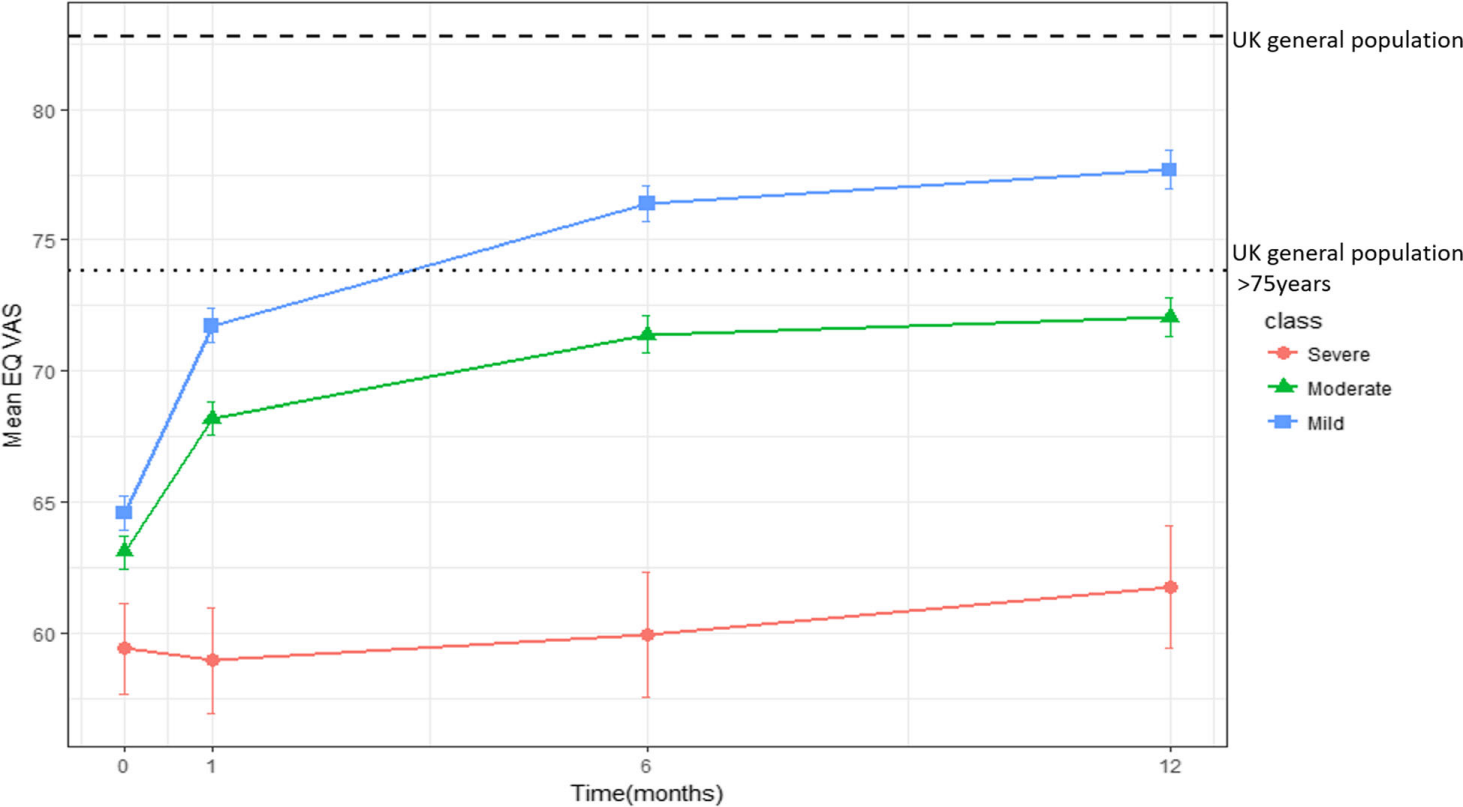
Temporal changes of HRQoL by multimorbidity class. The EQ-VAS scores range from 0 (worst) to 100 (best) health status with a difference of 7 points considered clinically meaningful. Patients with severe multimorbidity have worse health-related quality of life, shown by EQ-VAS scores, at baseline and through 12 months of follow-up

Table 2 shows descriptive statistics for EQ-VAS scores by multimorbidity clusters. Without adjustment for differences in patient characteristics, perceptions of health state measured by EQ-VAS scores were worse in the severe multimorbidity class compared to the mild and moderate classes suggesting better recovery of HRQoL in the mild and moderate multimorbidity classes compared to the severe class and these differences persisted at 12 months (Fig. 2). Compared with the moderate and mild classes, patients in the severe class had lower EQ-VAS scores at hospitalisation (59.39 vs. 63.08, 64.57), at 30 days (58.95 vs 68.20, 71.73), 6 months (59.92 vs 71.38, 76.39), and 12 months (61.73 vs 72.05, 77.69).

**Table 2:** EQ-VAS and EQ-5D utility scores by follow-up time and multimorbidity class

Variable	Multimorbidity class 1 (severe) 591 (6.5%)	Multimorbidity class 2 (moderate) 4301 (47.6%)	Multimorbidity class 3 (mild) 4147 (45.9%)	***P*** value
**EQ-5D, mean (SD)**				
Baseline	0.62 (0.31)	0.71 (0.29)	0.75 (0.28)	<0.001*
1 month	0.61 (0.30)	0.73 (0.26)	0.78 (0.24)	<0.001*
6 months	0.60 (0.30)	0.75 (0.26)	0.82 (0.24)	<0.001*
12 months	0.59 (0.30)	0.76 (0.27)	0.83 (0.24)	<0.001*
**Utilities: EQ-VAS, mean (SD)**				
Baseline	59.39 (20.83)	63.08 (20.30)	64.57 (21.01)	<0.001*
1 month	58.95 (19.36)	68.20 (18.27)	71.73 (17.73	<0.001*
6 months	59.92 (20.79)	71.38 (18.54)	76.39 (17.22)	<0.001*
12 months	61.73 (19.43)	72.05 (18.60)	77.69 (17.31)	0.001*
**EQ-5D dimensions baseline**				
Mobility	398 (67.34)	1833 (42.62)	1048 (25.27)	<0.001*
Self-care	163 (27.58)	690 (16.04)	399 (9.62)	<0.001*
Activities	370 (62.61)	2115 (49.17)	1837 (44.30)	<0.001*
Pain	288 (48.73)	1662 (38.64)	1178 (28.41)	<0.001*
Anxiety and depression	221 (37.39)	1473 (34.25)	1381 (33.30)	0.081
**EQ-5D 30 days**				
Mobility	264 (71.35)	1364 (43.86)	683 (24.03)	<0.001*
Self-care	120 (32.61)	481 (15.49)	215 (7.58)	<0.001*
Activities	275 (74.53)	1862 (60.06)	1498 (53.05)	<0.001*
Pain	228 (61.79)	1484 (47.81)	1077 (38.08)	<0.001*
Anxiety and depression	160 (43.72)	1152 (37.13)	1066 (37.54)	0.05
**EQ-5D 6 months**				
Mobility	209 (70.13)	1149 (43.31)	538 (23.08)	<0.001*
Self-care	97 (33.33)	387 (14.63)	173 (7.42)	<0.001*
Activities	216 (71.76)	1220 (46.02)	766 (32.90)	<0.001*
Pain	208 (69.57)	1280 (48.25)	808 (34.71)	<0.001*
Anxiety and depression	129 (43.14)	821 (30.99)	730 (31.32)	<0.001*
**EQ-5D 12 months**				
Mobility	207 (76.10	1080 (44.68)	477 (22.60)	<0.001*
Self-care	97 (35.27)	389 (16.11)	166 (7.88)	<0.001*
Activities	203 (74.09)	1091 (45.19)	571 (27.05)	<0.001*
Pain	195 (70.91)	1126 (46.94)	673 (32.08)	<0.001*
Anxiety and depression	99 (36.00)	696 (28.88)	568 (26.92)	0.006*

Values are given as the mean (standard deviation) for EQ-VAS, EQ-5D utility scores. Values are given as frequencies (percentages) for EQ-5D dimensions. *Significant after Hochberg correction using a false discovery rate of 0.05

### Multimorbidity and HRQoL measured by EQ-5D

Responses from the EQ-5D dimensions showed that during hospitalisation, 69.1% (6607/9566) of participants reported ≥ one problem across the EQ-5D dimensions, which increased to 73.9% (4935/6679) at 1 month, decreased to 62.6% (3491/5572) at 6 months, and 59.7% (3011/5047) at 12 months. Overall, the most frequently reported problems at baseline were for activities (50.1%), followed by mobility (37.6%), pain (35.5%), anxiety and depression (35.4%), and self-care (14.4%). At 12 months, they were pain (41.8%), followed by activities (38.8%), mobility (36.7%), anxiety and depression (28.6%), and self-care (13.6%). Table 2 shows descriptive statistics for EQ-5D scores by multimorbidity clusters.

Compared with the mild class, the percentage reporting ≥ 1 problem on the EQ-5D dimension at hospitalisation was higher in the severe and moderate classes (severe 85.3%; moderate 72.8% vs mild 65.5%), at 1 month (89.2%, 76.1% vs 69.0%), 6 months (85.9%, 67.5% vs 53.9%), and 12 months (88.6%, 65.3% vs 49.2%). At hospitalisation, individuals in the severe class were more likely to report having problems in all EQ-5D dimensions compared to the moderate and mild classes: mobility (severe 67.3% vs. mild 42.6%, mild 25.3%, respectively), self-care (27.6% vs 16.0%, 9.6%), activities (62.6% vs 49.2%, 44.3%), pain (48.7%% vs. 38.6%, 28.4%), anxiety and depression (37.4% vs 34.3%, 33.3%), and the prevalence of problems continued to be higher in the severe class at 1, 6, and 12 months. Individuals in the severe class were also likely to report problems with mobility, self-care, activities, pain, anxiety, and depression at 1, 6, and 12 months (Table 2). Compared with the moderate and mild classes, patients in the severe class had lower EQ-5D scores at hospitalisation (0.62 vs. 0.71, 0.75), at 30 days (0.61 vs 0.73, 0.78), 6 months (0.60 vs 0.75, 0.82), and 12 months (0.59 vs 0.76, 0.83).

### Adjusted results from multilevel modelling of longitudinal EQ-VAS, EQ-5D scores, and multimorbidity classes

Compared with the baseline, the adjusted average health state scores improved at 1 month (difference 4.62, 95% CI 4.04 to 5.19) and 6 months (3.43, 2.11 to 4.75), but declined at 12 months (−1.10, −3.55 to 1.35) suggesting a poorer health state at 12 months and greatest improvement at one month (Table 3). After adjusting for covariates, compared to the mild multimorbidity class, increased multimorbidity was associated with a significant decline in EQ-VAS scores (adjusted coefficient: −5.12 [95% CI −7.04 to −3.19] and −0.98 [−1.93 to −0.04] for severe and moderate multimorbidity, respectively. Similarly, increased multimorbidity was associated with a decline in EQ-5D scores (adjusted coefficient: −0.16 [95% CI −0.18 to −0.13] and −0.05 [−0.06 to −0.04] for severe and moderate multimorbidity classes, respectively (Additional file 1: Table S5).

**Table 3:** Adjusted parameter estimates from multilevel modelling of EQ-VAS scores and multi-morbidity classes, regression coefficient, and 95% confidence intervals

Variable	Regression coefficient (95% CI)	***P*** value
Intercept	48.39 (40.06 to 56.72)	
Month, baseline (ref)		
1 month	4.62 (4.04 to 5.19)	<0.001*
6 months	3.43 (2.11 to 4.75)	<0.001*
12 months	−1.10 (−3.55 to 1.35)	0.38
**Multimorbidity classes** Mild (ref)		
Moderate	−0.98 (−1.93 to −0.04)	0.04
Severe	−5.12 (−7.04 to −3.19)	<0.001*
Diagnosis(STEMI) ref		
Diagnosis (NSTEMI)	−0.26 (−1.19 to 0.65)	0.57
Age	0.12 (0.08 to 0.15)	<0.001*
Women	−4.17 (−5.02 to −3.32)	<0.001*
Ethnicity White	0.81 (−1.61 to 3.24	0.51
Ex/current smoking status	−1.07 (−1.84 to -0.30)	0.01
Previous MI	−1.37 (−2.64 to −0.10)	0.03
Previous Angina	−2.15 (−3.27 to −1.04)	<0.001*
**Treatments**		
Previous PCI	−1.53 (−2.96 to 0.11)	0.035
Previous CABG surgery	−3.03 (−4.64 to −1.42)	<0.001*

Adjusting for age, sex, ethnicity (white versus other) smoking status (never vs ex or current), past medical history of MI, angina, diagnosis (STEMI or NSTEMI), revascularisation (percutaneous coronary intervention [PCI] vs. no PCI; coronary artery bypass graft [CABG] surgery vs no CABG surgery), medications (B-blockers, statins, ACE, aspirin), cardiac rehabilitation (yes/no), and interactions of time and multimorbidity. Note: *CABG* coronary artery bypass grafting, *PCI* percutaneous coronary intervention, *MI* myocardial infarction, *STEMI* ST-elevation myocardial infarction, *NSTEMI* non-ST-elevation myocardial infarction, ^*^Significant after Hochberg correction using a false discovery rate of 0.05

### Adjusted results from multilevel modelling of longitudinal EQ-5D dimensions and multimorbidity classes

**Fig. 3:**
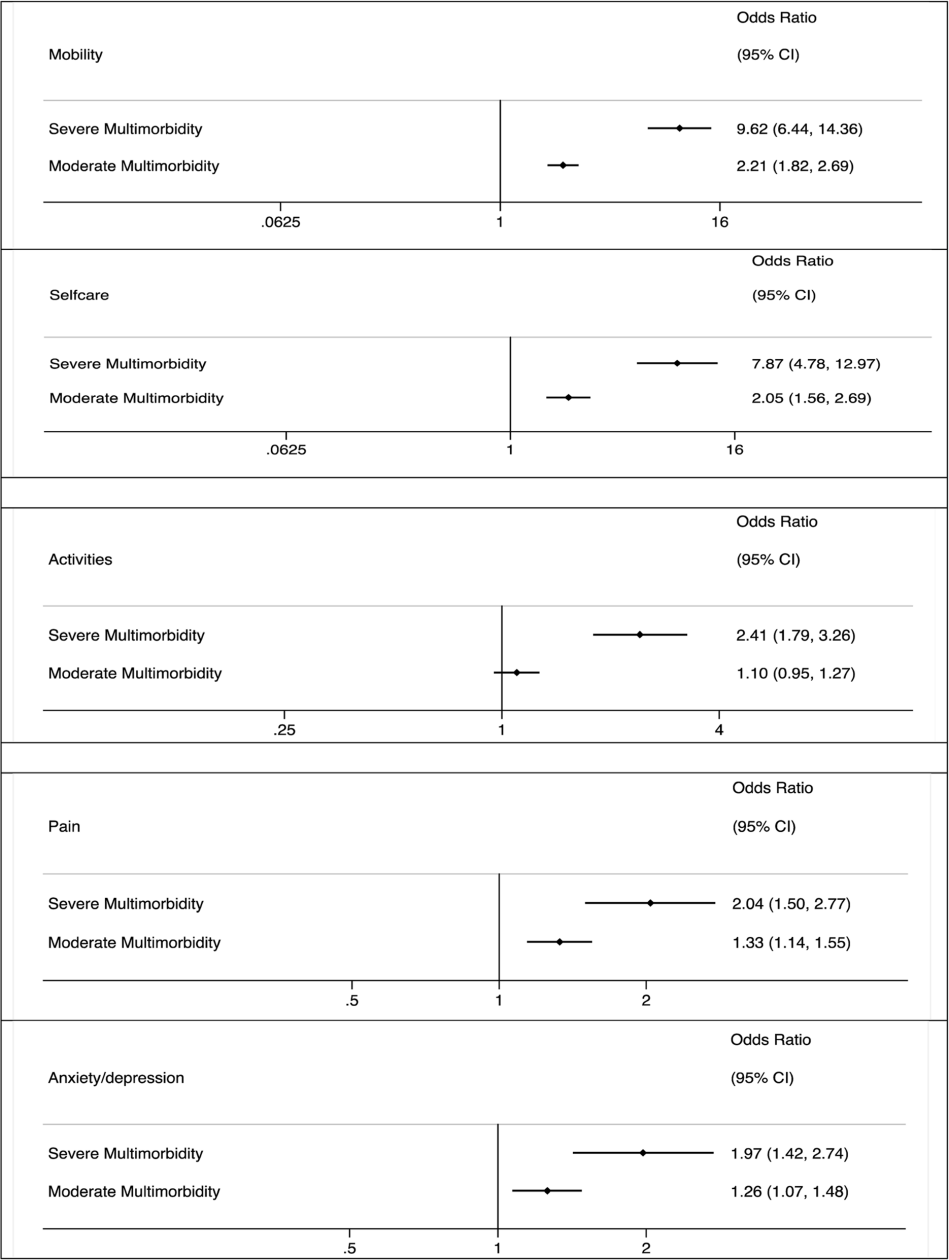
Association of multimorbidity classes and EQ-5D dimensions, (Mobility, Self-care, Activities, Pain and Anxiety/depression), odds ratios and 95% confidence intervals (reference group, mild multimorbidity). Adjusting for age, sex, ethnicity (white versus other) smoking status (never vs ex or current), past medical history of MI, angina, diagnosis (STEMI or NSTEMI), revascularisation (percutaneous coronary intervention [PCI] vs. no PCI; coronary artery bypass graft [CABG] surgery vs no CABG surgery), cardiac rehabilitation (yes/no) and interactions of time and multimorbidity, and medications

The multilevel logistic regression analysis results for the association of multimorbidity classes with five EQ-5D dimensions are shown in Fig. 3. After adjustment, individuals in the severe class were more likely than those in the mild class to report problems in mobility, OR 9.62 (95% confidence interval: 6.44 to 14.36), self-care 7.87 (4.78 to 12.97), activities 2.41 (1.79 to 3.26), pain 2.04 (1.50 to 2.77), and anxiety/depression 1.97 (1.42 to 2.74).

Similarly, individuals in the moderate class were more likely than those in the mild class to report problems with their mobility: 2.21 (1.82 to 2.69), self-care 2.05 (1.56 to 2.69), activities 1.10 (0.95 to 1.27), pain 1.33 (1.14 to 1.55), and anxiety/depression 1.26 (1.07 to 1.48).

## Discussion

In this nationwide study of 9566 patients with MI who were followed for 1 year, multimorbidity was common and associated with low HRQoL. Uniquely, our study identifies three distinct clusters of multimorbidity: severe (older/multiple chronic conditions), moderate (older/hypertension/diabetes), and mild (younger/low rates of chronic conditions). Different patterns of multimorbidity were associated with different HRQoL trajectories. We found an inverse relationship between extent of multimorbidity and improvement in HRQoL over time. Those with a high burden of multimorbidity at baseline had poor HRQoL, which failed to recover following MI. Notably, the EQ-VAS scores reported in patients with MI across all three multimorbidity classes were lower than those which have been reported in other common chronic conditions such as COPD [43] and heart failure [44], and UK general population [45]. Severe baseline multimorbidity was adversely associated with the EQ-5D dimensions mobility, self-care, activities, pain, anxiety, and depression. Indeed, severe multimorbidity was associated with an average decrease of 5 points in EQ-VAS scores compared to the mild multimorbidity cluster. Older MI survivors with high rates of multimorbidity, and patients with hypertension and diabetes are more likely to be associated with poor HRQoL.

Several previous studies have looked at the associations of individual chronic health conditions and health-related quality of life but a few focussed on combinations of disease clusters and their impact on health-related quality of life in MI survivors. Our study made a contribution to existing research by investigating the impact of different multimorbidity clusters on changes in HRQoL in MI survivors. Understanding disease clusters and their impact on HRQoL provides more insight for designing patient-centred care interventions. Our findings are consistent with previous studies that have found a decline in health-related quality of life in patients with hypertension, diabetes [46–48], and lung diseases [14, 19]. Such chronic conditions are related with stress, and their co-existence may accentuate the impact on psychological wellbeing owing to care demands [23]. Similar to other previous studies, we found a significant association between increased multimorbidity and anxiety and depression. This finding is consistent with previous research that reported an association between depression and cardiovascular diseases [49, 50]. Whilst it is known that a greater burden of comorbidities is associated with increased depression and other mental health conditions [1], the complex interplay between these remains unclear [50].

Our findings have similarities and differences from another study [51] that found five distinct multimorbidity patterns in Asian patients with heart failure. Discrepancies in number of classes between our study and that by Tromp et al. could be due to differences in the cohort health conditions. The study by Tromp et al. used a cohort of patients with heart failure, whilst our cohort were patients with myocardial infarction. Furthermore, we classified patients using 7 health conditions whilst the study by Tromp et al. used more than 7 health conditions including those that were not available in our study such as coronary artery disease, previous stroke, chronic kidney disease, peptic ulcer, cancer, liver disease, and dementia. Similar to our study, Tromp et al. identified a class with a high probability of having hypertension and diabetes and a class with low comorbidity rates.

Similar to our findings a UK study [4] using the same 7 health conditions that we used in our study found three distinct multimorbidity patterns in patients with myocardial infarction. There are similarities and differences in comorbidities combinations between our clusters and those found by Hall et al. The three multimorbidity patterns reported by Hall et al. were elderly with high multimorbidity (tended to have heart failure, PVD, and hypertension), medium multimorbidity patterns (tended to have PVD and hypertension), and younger with low multimorbidity (patients with few comorbidities). We also found an elderly high multimorbidity cluster with high comorbidities and a younger, low multimorbidity cluster with few comorbidities. However, the multimorbidity patterns were different between our medium multimorbidity cluster and that reported by Hall et al. The medium multimorbidity cluster reported in our study tended to have diabetes and hypertension whilst that reported by Hall et al. tended to have PVD and hypertension. These discrepancies could be due to different cohorts used by the two studies. Hall’s study used the MINAP registry which is much bigger (*n*=693,388) and more representative of the target population than the EMMACE cohort (*n*=9566) which comprises of a select cohort of patients who volunteered to participate in the study hence may exhibit selection bias. Furthermore, the EMMACE study excluded patients at a terminal stage of any illness, and those for whom follow-up was inappropriate or impractical. The study by Hall et al. had older patients with mean age 70.7 years compared to 64 years for the EMMACE study. Latent class analysis applied to a larger cohort may yield a different number of classes with different patterns [52]; therefore, there is need for more research to externally validate our study findings.

### Strengths and limitations

To our knowledge, this is the first study to map the distribution of different co-morbidity patterns among patients with MI and determine their impact on temporal changes in HRQoL using a large nationwide longitudinal cohort study. The breadth of the data increases generalisability, but with sufficient depth to allow multi-level modelling, and latent class analysis. We have extended previous knowledge by identifying distinct multimorbidity clusters and their impact on HRQoL in MI survivors. The EQ-5D captures important aspects of health status that may be affected by an MI, including mobility, activities, self-care, depression or anxiety, and pain. Whilst we recognise that using a disease-specific metric may have added additional insights, use of a generic tool allowed us to interpret the findings in the context of a range of other clinical conditions. Limitations of this work include the potential for participation bias and survivorship bias, in common with all such studies. In this study, multimorbidity classes were determined using seven health conditions that were recorded in the MINAP registry, more research using more health conditions is needed and to investigate whether the multimorbidity clusters change over time using latent transition models.

### Clinical implications

The identification of multimorbidity patterns and their impact on changes in HRQoL, and EQ-5D domains (mobility, activities, pain, anxiety, and depression) may help inform the design of targeted interventions [23], whereby therapies that better control co-morbidities in MI survivors may translate into meaningful improvement in HRQoL. Furthermore, patients with very high levels of multimorbidity might benefit from an early multidisciplinary team involvement to directly improve specific domains of their HRQoL. Multidisciplinary efforts to effectively manage cardiovascular risk factors (particularly diabetes and hypertension) may have an additional secondary preventive role.

## Conclusion

This multi-centre longitudinal study of over 9500 survivors of hospitalised MI found that multimorbidity was adversely associated with dimensions of HRQoL including mobility, self-care, and activities of daily living, pain, anxiety, and depression. Moreover, a data-driven approach has enabled the identification of three clinically distinct multimorbidity clusters with significant differences in HRQoL and who may be suitable for tailored interventions to improve and maintain their HRQoL following MI. Older survivors of MI with high rates of multimorbidity, and patients with hypertension and diabetes are more likely to be associated with poor HRQoL. As such, we recommend specific interventions to target these subgroups to improve their HRQoL.

## Supplementary Information

**Additional file 1.** Association of multimorbidity and changes in health related quality of life following myocardial infarction: A UK multicentre longitudinal patient-reported outcomes study. Figure S1-[Number of patients who enrolled in EMMACE 3 and 4]. Figure S2-[Normal probability plots for level 1 and level 2 residuals for (A) EQVAS and (B) EQ5D models]. Table S1-[latent class analysis model selection goodness of fit statistics]. Table S2-[Proportions of latent class based on their most likely latent class membership]. Table S3-[Class conditional probabilities of responses to the 7 comorbidities]. Table S4-[Comparison of baseline characteristics between respondent and non-respondents at 12 months]. Table S5-[Adjusted parameter estimates from multilevel modelling of EQ-5D scores and multi-morbidity classes, regression coefficient and 95% confidence intervals].

## Data Availability

The PIs of the EMMACE cohort datasets that were used in this study are not able to share individual level data due to ethical reasons. Additional related documents can be requested through the corresponding author of this manuscript.

## Abbreviations

HRQoL: Health-related quality of life
COPD: Chronic obstructive pulmonary disease
EMMACE: Evaluation of the Methods and Management of Acute Coronary Events
MI: Myocardial infarction
STEMI: ST-elevation myocardial infarction
NSTEMI: Non-STEMI
NHS: National Health Service
EQ-5D-3L: EuroQol 5-dimension 3-levels
MINAP: Myocardial Ischaemia National Audit Project
CVSD: Cerebrovascular disease
PVD: Peripheral vascular disease
EQ-VAS: Visual analogue scale
PCI: Percutaneous coronary intervention
CABG: Coronary artery bypass graft
B-Blockers: Beta-blockers
ACE: Angiotensin-converting enzyme
IMD: Index of Multiple Deprivations
