# Supplementary material for: Association of multimorbidity and changes in health-related quality of life following myocardial infarction: a UK multicentre longitudinal patient-reported outcomes study
Source: BMC Med. 2021 Sep 28;19:227. doi: 10.1186/s12916-021-02098-y (PMC8477511; doi:10.1186/s12916-021-02098-y)
Supplement: Supplementary file 1 — Additional file 1. Association of multimorbidity and changes in health related quality of life following myocardial infarction: A UK multicentre longitudinal patient-reported outcomes study. Figure S1-[Number of patients who enrolled in EMMACE 3 and 4]. Figure S2-[Normal probability plots for level 1 and level 2 residuals for (A) EQVAS and (B) EQ5D models]. Table S1-[latent class analysis model selection goodness of fit statistics]. Table S2-[Proportions of latent class based on their most likely latent class membership]. Table S3-[Class conditional probabilities of responses to the 7 comorbidities]. Table S4-[Comparison of baseline characteristics between respondent and non-respondents at 12 months]. Table S5-[Adjusted parameter estimates from multilevel modelling of EQ-5D scores and multi-morbidity classes, regression coefficient and 95% confidence intervals]. [file 12916_2021_2098_MOESM1_ESM.pdf]

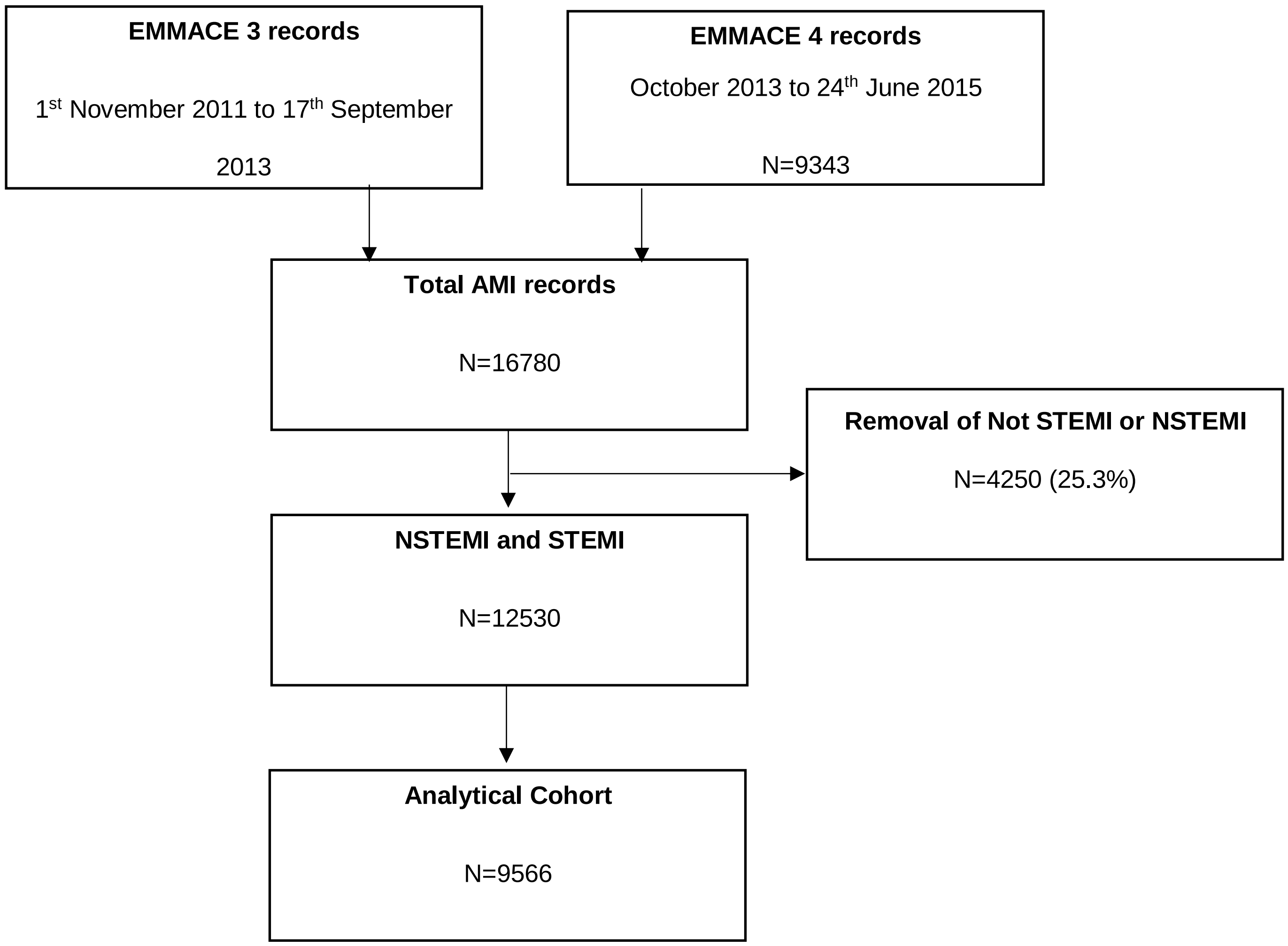

Figure S1: Number of patients who enrolled in EMMACE 3 and 4.

Table S1: latent class analysis model selection goodness of fit statistics

	**AIC**	**ssBIC**	**BLRT p value**	**Entropy**
1 class	39085.29	39113.11		
2 classes	38037	38096.65	<0.001	0.49
3 classes	37989.26	38080.67	<0.001	0.54
4 classes	37988.43	38111.65	0.19	0.70
5 classes	37990.24	38145.24	0.39	0.74

Table S2: Proportions of latent class based on their most likely latent class membership

**Final class counts and proportions for the latent class based on their most likely latent class membership**	**Average Latent Class Probabilities for Most Likely Latent Class Membership (Row) by Latent Class (Column)**
Latent Classes<br>1 591 0.06538<br>2 4301 0.47583<br>3 4147 0.45879	1 2 3<br>1 **0.810** 0.183 0.007<br>2 0.110 **0.739** 0.150<br>3 0.006 0.184 **0.810**

Table S3: Class conditional probabilities of responses to the 7 comorbidities

**Item**	**Class1**	**Class 2**	**Class 3**
PVD	0.14	0.04	0.008
CVSD	0.15	0.07	0.002
COPD	0.22	0.15	0.09
CRF	0.22	0.02	0.002
Heart failure	0.21	0.00	0.002
Diabetes	0.53	0.24	0.04
Hypertension	0.68	0.67	0.16

Note: CRF, Chronic Renal failure; PVD, Peripheral Vascular Disease; COPD, Chronic Obstructive Pulmonary Disease; Cerebrovascular disease CVSD.

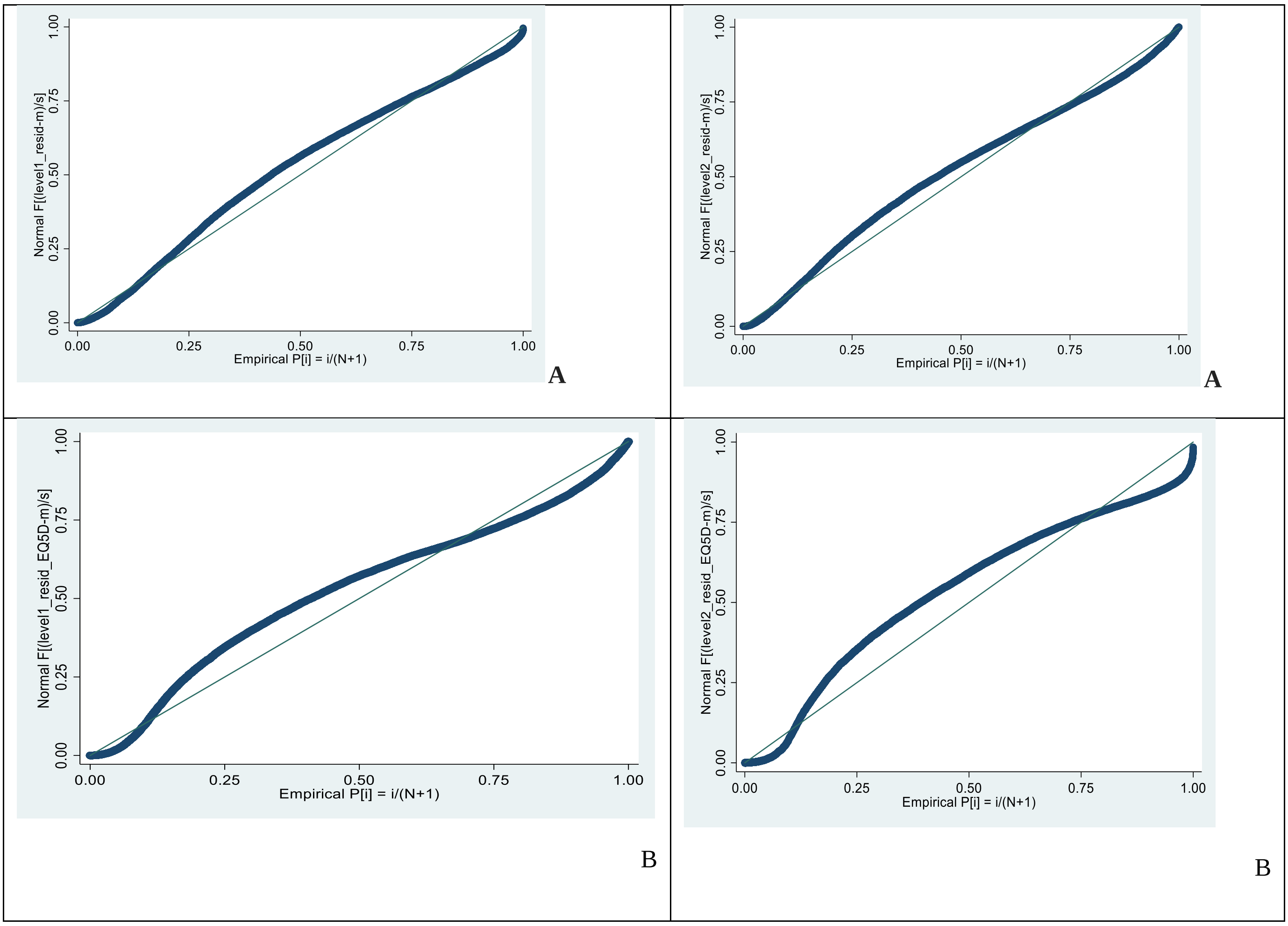

Figure S2: **Normal probability plots for level 1 and level 2 residuals for (A) EQVAS and (B) EQ5D models.**

Table S4: Comparison of baseline characteristics between respondent and non-respondents at 12 months

Variable	Respondents n=5047(52.8%)	Non respondents n=4519(47.2%)	Difference (95% CI)
Female, n (%)	1237(24.9)	1160(25.3)	-0.004(-0.02 to 0.01)
Age, mean(SD), year	65.9(10.8)	62.1(12.7)	3.89(3.42 to 4.37)
STEMI, n (%)	1965(39.5)	1943(42.4)	-0.03(-0.05 to -0.01)
NSTEMI, n (%)	3014(60.5)	2644(57.6)	0.03(0.01 to 0.05)
White ethnicity, n (%)	4251(85.4)	3885(84.7)	0.007(-0.007 to 0.02)
IMD, mean (SD)	21.29(14.8)	25.72(16.6)	-4.43(-5.39 to -3.48)
Previous angina, n (%)	938(18.8)	854(18.6)	0.002(-0.01 to 0.02)
Diabetes, n (%)	749(15.0)	965(21.0)	-0.06(-0.07 to -0.04)
Hypertension, n (%)	2174(43.7)	1904(41.5)	0.02(0.002 to 0.04)
Heart failure, n (%)	88(1.8)	124(2.7)	-0.01(-0.01 to -0.003)
PVD, n (%)	150(3.0)	167(3.6)	-0.01(-0.01 to 0.001)
CVSD, n (%)	202(4.1)	226(4.9)	-0.01(-0.02 to -0.001)
CRF, n (%)	136(2.7)	153(3.3)	-0.01(-0.01 to 0.001)
COPD, n (%)	574(11.5)	592(12.9)	-0.01(-0.03 to -0.001)
Ex/current smoker, n (%)	3014(60.5)	3234(70.5)	-0.1(-0.12 to -0.08)
Previous AMI, n (%)	733(14.7)	789(17.2)	-0.02(-0.04 to -0.01)
Previous PCI, n (%)	434(8.7)	465(10.1)	-0.01(-0.02 to -0.002)
CABG surgery, n (%)	333(6.7)	10(6.8)	-0.001(-0.001 to 0.01)
Baseline EQ-5D, mean(SD)	0.74(0.3)	0.70(0.3)	0.04(0.03 to 0.06)
Baseline EQ-VAS	64.5(20.4)	62.1(21.2)	2.37(1.52 to 3.22)

Note: IMD indicates Index of Multiple Deprivation; CABG, coronary artery bypass grafting; PCI, Percutaneous Coronary Intervention; AMI, Acute Myocardial Infarction; CRF, Chronic Renal failure; PVD, Peripheral Vascular Disease; COPD, Chronic Obstructive Pulmonary Disease; Cerebrovascular disease CVSD; Body Mass Index (BMI); ST-elevation myocardial infarction STEMI, non ST-elevation myocardial infarction NSTEMI.

Table S5: Adjusted parameter estimates from multilevel modelling of EQ-5D scores and multi-morbidity classes, regression coefficient and 95% confidence intervals

**Variable**	**Regression coefficient (95% CI)**	**P value**
Intercept	0.59(0.48 to 0.70)	
Month, Baseline (ref)		
1 month	0.01(0.001 to 0.01)	0.02
6 months	0.03(0.02 to 0.04)	<0.001*
12 months	0.04(0.03 to 0.05)	<0.001*
**Multimorbidity classes**<br>Mild(ref)		
Moderate	-0.05(-0.06 to -0.04)	<0.001*
Severe	-0.16(-0.18 to -0.13)	<0.001*
Diagnosis(STEMI) ref		
Diagnosis (NSTEMI)	-0.003(-0.02 to 0.01)	0.65
Age	0.001(0.001 to 0.002)	<0.001*
Women	-0.07(-0.08 to -0.06)	<0.001*
Ethnicity white	0.05(0.01 to 0.08)	0.01
Ex/current smoking status	-0.03(-0.04 to -0.02)	<0.001*
**Treatments**		
Previous PCI	-0.03 (-0.05 to -0.02)	<0.001*
Previous CABG surgery	-0.03 (-0.06 to -0.01)	0.003*

Adjusting for age, sex, ethnicity (white versus other) smoking status (never vs ex or current), angina, diagnosis (STEMI or NSTEMI), revascularisation (percutaneous coronary intervention [PCI] vs. no PCI; coronary artery bypass graft [CABG] surgery vs no CABG surgery), medications (B-Blockers, statins, ACE, Aspirin), cardiac rehabilitation (yes/no). Note: CABG, coronary artery bypass grafting; PCI, Percutaneous Coronary Intervention; MI, Myocardial Infarction, ST-elevation myocardial infarction STEMI, non ST-elevation myocardial infarction NSTEMI. *Significant after Hochberg correction using a false discovery rate of 0.05
